# 全血EB病毒DNA载量对异基因造血干细胞移植后淋巴增殖性疾病的诊断价值

**DOI:** 10.3760/cma.j.issn.0253-2727.2021.11.004

**Published:** 2021-11

**Authors:** 燕燕 牛, 玉君 董, 玥 尹, 蔚林 许, 赜隐 梁, 倩 王, 渊 李, 微 刘, 晋平 欧, 汉云 任

**Affiliations:** 1 北京大学第一医院血液科，北京 100034 Department of Hematology, Peking University First Hospital, Beijing 100034, China; 2 山西白求恩医院山西医学科学院血液科，太原 030032 Department of Hematology, Shanxi Bethune Hospital, Shanxi Academy of Medical Sciences, Taiyuan 030032, China

**Keywords:** 淋巴组织增殖性疾病, 异基因造血干细胞移植, EB病毒再活化, Lymphoproliferative disorders, Allogeneic hematopoietic stem cell transplantation, EBV reactivation

## Abstract

**目的:**

探讨全血定量PCR法检测EB病毒（EBV）DNA载量对于异基因造血干细胞移植（allo-HSCT）后淋巴增殖性疾病（PTLD）的诊断价值。

**方法:**

对2004年4月至2019年4月于北京大学第一医院血液科行allo-HSCT的694例血液病患者进行回顾性分析。

**结果:**

①694例allo-HSCT患者中29例（4.2％）发生PTLD，其中男22例，女7例，中位年龄22（1～52）岁，中位发病时间为移植后2.1（0.8～20.6）个月。②单因素分析显示年龄<30岁、再生障碍性贫血、HLA配型不合、预处理方案中含有ATG、EBV再活化是PTLD发生的危险因素，多因素分析显示EBV再活化为PTLD发生的独立危险因素。③对于EBV再活化病例进一步分析发现PTLD组中位EBV-DNA载量峰值明显高于非PTLD组（*P*<0.001），且随EBV-DNA拷贝增高PTLD发生率有增高的趋势。ROC曲线分析提示当EBV-DNA载量>1.19×10^6^拷贝/ml时诊断PTLD的可能性较大（灵敏度为0.800，特异度为0.768）。④全部PTLD病例均接受以利妥昔单抗为基础的治疗，总反应率为86.2％，总生存率为54.3％。

**结论:**

allo-HSCT后PTLD的发生与EBV再活化高度相关，EBV-DNA载量越高发生PTLD的风险越大，动态监测EBV-DNA载量对预测PTLD发生有重要作用。

## Abstract (en)

**Objective:**

To investigate the diagnostic value of whole blood quantitative PCR for DNA load of Epstein-Barr virus (EBV) in post-transplant lymphoproliferative disease (PTLD) after allogeneic hematopoietic stem cell transplantation (allo-HSCT).

**Methods:**

A total of 694 patients with hematologic diseases who underwent allo-HSCT at the Hematology Department of Peking University First Hospital from April 2004 to April 2019 were included, and their data were retrospectively analyzed.

**Results:**

①Among the 694 cases, 29 cases (22 males and 7 females, with a median age of 22 (1–52) years) developed PTLD after allo-HSCT with a cumulative incidence of 4.2％ and a median onset time of 2.1 (0.8–20.6) months. ② Univariate analysis showed that age<30 years, diagnosis with aplastic anemia, human leukocyte antigen (HLA) mismatch, use of antithymocyte globulin (ATG) in preconditioning regimens, and EBV reactivation were the risk factors for the occurrence of PTLD. Multivariate analysis showed that EBV reactivation was an independent risk factor for the occurrence of PTLD. ③Further analysis of EBV reactivation cases showed that the peak value of EBV-DNA load was significantly higher in the PTLD group than that in the non-PTLD group (*P*<0.001) and the incidence of PTLD increased with the increase of EBV-DNA load. Receiver operating characteristic (ROC) curve analysis indicated that PTLD was more likely to be diagnosed when the EBV-DNA load was >1.19×10^6^ copies/ml (sensitivity 0.800 and specificity 0.768). ④All patients with PTLD received rituximab-based treatment, with an overall response rate of 86.2％ and an overall survival rate of 54.3％.

**Conclusion:**

The PTLD occurrence after allo-HSCT is highly correlated with EBV reactivation, and the higher the EBV-DNA load, the greater the risk of PTLD occurrence. The dynamic monitoring of EBV-DNA load plays an important role in predicting PTLD occurrence.

淋巴增殖性疾病（PTLD）是异基因造血干细胞移植（allo-HSCT）后一种严重的并发症，是机体免疫功能低下时淋巴细胞增殖而来的异质性淋巴增殖性病变[1]–[2]，发生率约为4％[3]，近年来发病率呈上升趋势。文献报道PTLD发生的危险因素包括T细胞去除、HLA不相合/无关供者移植、脐血干细胞移植、供受者EB病毒（EBV）状态、减低预处理剂量、移植物抗宿主病和原发性免疫缺陷病等，危险因素越多PTLD发生率越高[4]–[5]。allo-HSCT后PTLD几乎均由EBV再活化所致[6]–[7]。EBV特异性细胞毒性T淋巴细胞（EBV-CTL）治疗PTLD疗效确切，但临床应用困难。本研究回顾性分析了我院allo-HSCT患者PTLD的发生与EBV再活化及EBV-DNA负荷的关系，为临床进一步提高对该疾病的认识提供经验参考。

## 病例与方法

一、病例资料

本研究纳入自2004年4月至2019年4月在北京大学第一医院血液科行allo-HSCT的694例血液病患者，其中男406例，女288例，中位年龄31（1～69）岁。疾病类型：急性髓系白血病（AML）277例，急性淋巴细胞白血病（ALL）151例，骨髓增生异常综合征（MDS）83例，慢性髓性白血病（CML）52例，重型再生障碍性贫血（SAA）36例，淋巴瘤66例，浆细胞疾病14例，其他疾病15例。HLA全相合同胞供者移植175例，HLA不相合亲缘供者移植409例，HLA非血缘供者移植110例。预处理方案包含抗胸腺细胞球蛋白（ATG）435例，不含ATG 259例。

二、预处理方案

AML、MDS、CML采用白消安（Bu）/环磷酰胺（Cy）±ATG或Bu/氟达拉滨（Flu）±ATG为基础的方案；ALL采用依托泊苷（Vp16）+Bu/Cy±ATG或含全身照射（TBI）为主的方案；非霍奇金淋巴瘤（NHL）和多发性骨髓瘤采用BEAM为主的方案；SAA采用Flu/Cy+ATG为主的方案。ATG应用剂量为7.5～10 mg/kg。

三、GVHD的预防、诊断和治疗

GVHD预防采用环孢素A（CsA）+霉酚酸酯（MMF）+短程甲氨蝶呤（MTX）为主的方案，CsA谷浓度维持150～250 µg/L并根据GVHD发生情况和患者原发病情况进行个体化调整，移植4个月后逐渐减少CsA用量。发生在移植后100 d内的GVHD称为急性GVHD，依据最新修订的NIH标准进行诊断。234例患者发生急性GVHD（Ⅰ/Ⅱ度131例，Ⅲ/Ⅳ度103例），460例患者未发生GVHD。

四、移植后造血重建的判定

粒细胞植入：中性粒细胞绝对计数（ANC）>0.5×10^9^/L连续3 d；血小板植入：血小板计数>20×10^9^/L连续7 d且脱离血小板输注。以骨髓细胞遗传学检查、ABO血型检测、供受者嵌合度检测证实供者植入情况。

五、病毒预防与监测

在预处理阶段应用更昔洛韦（5 mg/kg每12 h 1次，连续7 d～10 d）进行病毒预防，移植后常规口服阿昔洛韦（0.4 g每日2次）1年以上。移植后早期每周1次应用定量PCR方法进行全血EBV-DNA监测，EBV活化指标为EBV-DNA>500 拷贝/ml。以诊断PTLD时全血EBV-DNA水平和非PTLD患者EBV-DNA最高水平进行比较。192例患者移植后发生EBV活化，502例未发生EBV活化。

六、PTLD的诊断标准

PTLD的诊断包括临床诊断和确定诊断，病理学分型依据WHO2016标准。临床诊断标准：①具有HLA不相合供者、移植物去除T细胞、使用ATG、单抗及其他免疫抑制剂等高危因素；②移植后出现经验性抗生素治疗无效的发热、肝脾及淋巴结肿大等临床表现或相应影像学表现，伴EBV拷贝数增加。确定诊断除包括以上临床诊断标准外，还包括病理活检证实病变具有PTLD的特征（以器官侵袭和淋巴结结构破坏为特点的各种弥漫性淋巴增殖性改变、原位杂交阳性）。病理学将PTLD分为4型：①早期病变：包括反应性浆细胞增生和传染性单核细胞增多症样的PTLD；②多形性PTLD；③单形性PTLD：包括T、B细胞淋巴瘤；④霍奇金淋巴瘤或霍奇金淋巴瘤样PTLD。

七、随访

随访截止日期为2020年12月31日，中位随访时间8.4（0.1～60.5）个月。通过查阅患者住院病历、门诊随访和电话随访方式进行随访。

八、统计学处理

采用SPSS25.0进行数据分析，疾病发生率的比较采用*χ*^2^检验或Fisher确切概率法分析，两组EBV拷贝数之间比较采用非参数检验，单因素分析中*P*<0.05的变量纳入多因素分析，采用logistic回归分析影响PTLD发生的独立危险因素。*P*<0.05认为差异有统计学意义。应用ROC曲线寻找EBV载量在诊断PTLD时最佳临界点。

## 结果

一、PTLD发生及相关因素分析

694例allo-HSCT患者中有29例（4.2％）发生PTLD，其中男22例，女7例，中位年龄22（1～52）岁，中位发病时间为移植后2.1（0.8～20.6）个月。对性别、年龄、原发病、HLA配型情况、移植物来源、预处理中是否包含ATG、EBV感染情况、移植后GVHD情况进行单因素分析比较。结果显示：不同性别间PTLD发生率差异无统计学意义（*P*＝0.053）；年龄<30岁组PTLD发生率为6.15％（20例），高于年龄≥30岁组（2.44％，9例）（*P*＝0.015）；不同疾病类型PTLD发生率差异有统计学意义（*P*＝0.001），其中SAA组PTLD发病率为19.44％，高于其他组；不同HLA配型组PTLD发生率不同（*P*＝0.023），其中同胞不合供者组发生率5.87％，高于其他两组；供受者性别组间差异无统计学意义（*P*＝0.127）；不同移植物来源组PTLD发生率差异无统计学意义（*P*＝0.628）；预处理方案中包含ATG组PTLD患者（28例）发生率为6.44％，高于不含ATG组患者（1例）发生率0.39％，差异有统计学意义（*P*<0.001）；EBV再活化组PTLD发生率为14.58％（28例）明显高于EBV未活化组（0.2％，1例）（*P*<0.001）；Ⅰ/Ⅱ度急性GVHD组与Ⅲ/Ⅳ度急性GVHD组间PTLD发生率无统计学意义（*P*＝0.917）（表1）。多因素分析显示EBV感染为PTLD发生的独立危险因素（表1）。

**表1:** 694例血液病患者异基因造血干细胞移植（allo-HSCT）后淋巴增殖性疾病（PTLD）相关危险因素分析［例（％）］

因素	未发生PTLD（665例）	PTLD（29例）	单因素分析		多因素分析	
			*χ*^2^值	*P*值	*OR*（95%*CI*）	*P*值
性别			3.757	0.053		
男	384（94.58）	22（5.42）				
女	281（97.57）	7（2.43）				
年龄			5.956	0.015	0.627（0.264～1.491）	0.291
<30岁	305（93.85）	20（6.15）				
≥30岁	360（97.56）	9（2.44）				
诊断			15.690	0.001	1.080（0.876～1.330）	0.471
AML+MDS+CML	400（97.09）	12（2.91）				
ALL	143（94.70）	8（5.30）				
SAA	29（80.56）	7（19.44）				
其他	93（97.89）	2（2.11）				
供者类型			7.522	0.023	0.890（0.409～1.973）	0.790
同胞HLA全相合	173（98.86）	2（1.14）				
亲缘HLA不全相合	385（94.13）	24（5.87）				
无关供者	107（97.27）	3（2.73）				
供者/患者性别			5.700	0.127		
女/男	151（95.57）	7（4.43）				
女/女	117（99.15）	1（0.85）				
男/男	232（93.93）	15（6.07）				
男/女	165（96.49）	6（3.51）				
干细胞来源			0.931	0.628		
外周血	109（97.32）	3（2.68）				
骨髓	524（95.45）	25（4.55）				
骨髓+外周血	32（96.97）	1（3.03）				
应用ATG			14.844	<0.001	7.571（0.983～58.295）	0.052
否	258（99.61）	1（0.39）				
是	407（93.56）	28（6.44）				
EBV活化			71.765	<0.001	60.939（8.168～454.660）	<0.001
否	501（99.80）	1（0.20）				
是	164（85.42）	28（14.58）				
急性GVHD						
Ⅰ/Ⅱ度	566（95.77）	25（4.23）	0.011	0.917		
Ⅲ/Ⅳ度	99（96.12）	4（3.88）				

注：AML：急性髓系白血病；ALL：急性淋巴细胞白血病；MDS：骨髓增生异常综合征；CML：慢性髓性白血病；SAA：重型再生障碍性贫血；ATG：抗胸腺细胞球蛋白；EBV：EB病毒

二、PTLD的临床及病理特征

29例PTLD患者中，26例发生于移植后6个月内，其余3例分别发生于移植后9、13、20个月。其中15例患者出现反复高热，影像学检查及微生物学检查无细菌和真菌感染证据，经验性广谱抗生素抗感染治疗无效；26例患者出现淋巴结肿大，多数为颈部、锁骨上、腋窝等浅表淋巴结；1例出现肝脏、胰尾占位，多发骨质破坏，伴腹膜后多发淋巴结肿大；1例淋巴结肿大伴脾肿大；1例表现为发热伴肺内结节；2例表现为噬血细胞综合征，伴心功能不全、肾功能不全；4例合并消化道出血；4例合并出血性膀胱炎。所有患者均伴有不同程度的血细胞减少。

在29例PTLD患者中，14例为临床诊断，15例行病理检查确诊，单形性PTLD 14例（包括弥漫大B细胞淋巴瘤9例、浆细胞肿瘤5例），多形性PTLD 1例。14例免疫组化示CD20++～+++，1例单形性PTLD（浆细胞肿瘤）CD20阴性，15例Ki67为60％～95％阳性。所有病理诊断组织采用EBER原位杂交技术检测，除1例单形性PTLD（浆细胞肿瘤）EBER阴性外，其余均阳性。

三、EBV再活化患者EBV-DNA负荷与PTLD发生的关系

由于allo-HSCT后PTLD发生几乎全部与EBV再活化相关，我们分析了2010年后进行规律定量检测EBV-DNA负荷的病例共542例，发现EBV再活化者为167例，再活化率为30.8％，其中142例患者未发生PTLD，25例发生PTLD，PTLD发生率为15.0％。在142例EBV再活化但未发生PTLD的病例中，EBV-DNA载量峰值［*M*（*P*_25_,*P*_75_）］为1.23×10^5^（1.53×10^4^，8.70×10^5^）拷贝/ml，而在25例发生PTLD患者全部为EBV再活化病例，诊断时EBV-DNA载量［*M*（*P*_25_,*P*_75_）］为3.20×10^6^（1.44×10^6^，7.47×10^6^）拷贝/ml，两组EBV-DNA载量差异有统计学意义（*z*＝−4.362，*P*<0.001）。进一步将不同EBV-DNA载量分组进行比较，结果显示随着EBV-DNA载量的增加，PTLD发生率逐步增加，其中10^6^～10^7^及以上组PTLD发生率明显增高（*P*<0.001）（表2）。

**表2:** 不同EB病毒DNA载量组移植后淋巴增殖性疾病（PTLD）发生率的比较

组别	例数	EB病毒DNA载量（拷贝/ml）			
		<10^4^	10^4^～10^5^	10^6^～10^7^	>10^7^
未发生PTLD组（142例）	142	26（96.30）	82（95.35）	34（64.15）	0（0.00）
PTLD组（25例）	25	1（3.70）	4（4.65）	19（35.85）	1（100.00）

*χ*^2^值		33.717			
*P*值		<0.001			

针对EBV-DNA峰值载量构建ROC曲线，判断其对于PTLD组和未发生TLD组的预测价值，结果显示当EBV载量为1.19×10^6^拷贝/ml时，ROC曲线下面积最大（0.774），灵敏度为0.800，特异度为0.768，提示当EBV-DNA载量大于1.19×10^6^拷贝/ml时，发生PTLD的可能性较大（图1）。

**图1:**
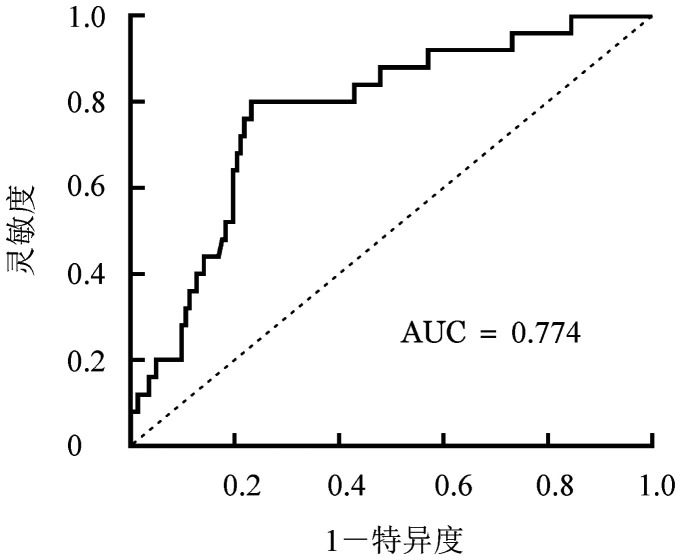
ROC曲线分析EBV-DNA载量诊断PTLD的敏感性和特异性 EBV：EB病毒；PRLD：淋巴增殖性疾病

四、PTLD的治疗与随访情况

29例患者于诊断后给予不同程度减停免疫抑制剂，阿昔洛韦、更昔洛韦、膦甲酸钠单药或联合抗病毒治疗，24例联合应用利妥昔单抗单药（375 mg/m^2^每周1次，中位4次），2例联合应用利妥昔单抗+静脉免疫球蛋白，1例联合应用利妥昔单抗+供者淋巴细胞输注（DLI），1例联合应用利妥昔单抗+EBV-CTL，1例联合应用利妥昔单抗+化疗+EBV-CTL。25例（86.2％）患者对治疗有反应，表现为体温控制、不同程度的淋巴结缩小和EBV拷贝数下降或转阴。对治疗有反应患者全血EBV-DNA拷贝数明显低于治疗无反应组（*z*＝−3.036，*P*<0.05）。至随访截止，13例存活，12例死亡（其中7例患者后期死于感染，4例死于原发病复发，1例死于颅内出血），4例失访，总生存率为54.3％，死亡患者与存活患者间EBV-DNA拷贝数无明显差异（*P*＝0.129）。

## 讨论

PTLD是allo-HSCT的致命合并症之一，发展迅速，死亡率高，近年来逐渐受到重视。其特点是EBV感染所致淋巴细胞转化而发生不受控制的淋巴细胞增殖。EBV再激活和免疫监视受损是PTLD发病机制中的关键因素[8]。EBV是一种广泛存在的双链DNA疱疹病毒，90％以上成人曾被感染[9]。移植受者体内潜伏的EBV可为受者或供者来源，在移植后免疫抑制的状态下重新激活。一方面，在没有适当的免疫监视且免疫功能破坏的情况下，潜伏于B细胞中的EBV可在生发中心长期存在，致使原癌基因异常造成体细胞超突变、BCL-6和c-MYC表达改变，激活NF-κB、PI3K/AKT/mTOR和BCL2通路，出现异常免疫球蛋白类型转换，最终导致淋巴组织增生，从早期病变向多形性PTLD转变，后逐步转化为单形性PTLD[10]–[12]。目前尚无相关报导EBV-DNA载量与PTLD病理类型之间的相关性。在本研究中，15例患者经病理确诊，其中14例EBER检测阳性，弥漫大B细胞淋巴瘤9例，浆细胞肿瘤4例，未发现EBV-DNA载量与病理类型的关系。另一方面，免疫抑制降低了T细胞的数量和质量，无法产生干扰素γ、白细胞介素2和肿瘤坏死因子α等细胞因子[13]。慢性持续的EBV血症可致T淋巴细胞上PD-1等共抑制受体PD-L1上调，当与PD-1受体结合时，使EBV特异性的T细胞失活[14]–[15]。EBV潜伏蛋白还可以通过下调主要组织相容性复合体Ⅰ和Ⅱ表达来逃避免疫监视[16]。

PTLD的发生与多项危险因素有关。日本一项多中心回顾性研究分析了64 539例移植患者，在多因素分析中发现预处理方案中包含ATG、应用ATG治疗急性GVHD、HLA全相合亲缘供者以外的移植、再生障碍性贫血患者，二次allo-HSCT以及伴发急性GVHD为PTLD发生的危险因素。再生障碍性贫血患者和脐血干细胞移植患者的PTLD发生率增高可能与应用ATG有关。ATG被认为是PTLD发生的一个重要危险因素，T细胞耗竭可以使不同器官EBV感染的B细胞增殖和浸润，高剂量ATG导致PTLD风险比低剂量ATG增加2.3倍[17]–[18]。Kanakry等一项研究纳入785例单倍型或无关供者造血干细胞移植患者，采用后置环磷酰胺（PTCy）方案预防GVHD，所有患者均未发生PTLD，且没有增加GVHD的发生率[19]。另一项研究显示在非血缘脐血干细胞移植中，以含ATG非清髓方案进行预处理的患者具有较高的PTLD发生率 [20]。急性GVHD患者促炎症细胞因子导致细胞毒性T细胞和T辅助细胞功能下降，T细胞损伤可能增加PTLD的发生风险[21]。再生障碍性贫血患者经免疫抑制治疗后，EBV活化率较高，同时应用含ATG预处理可能是其PTLD高发的原因[22]–[23]。本研究发现患者年龄<30岁、预处理方案包含ATG、原发病为再生障碍性贫血、HLA配型不合、EBV感染是PTLD发生的危险因素，这与Fujimoto等的回顾性分析结果基本一致[18]。但本研究并未发现伴发急性GVHD与PTLD发生相关，可能与GVHD的治疗措施不同有关。多因素分析结果表明EBV再活化是PTLD发生的独立危险因素。本研究29例发生PTLD患者中28例EBV阳性，与非PTLD组比较EBV-DNA载量明显增高，且随着病毒载量的增加，PTLD发生率逐步增高，经ROC曲线分析 EBV-DNA载量>1.19×10^6^拷贝/ml时发生PTLD可能性明显增加。EBV活化与免疫抑制的强度可能有密切相关性。免疫抑制越严重，EBV活化越明显，EBV-DNA载量越大。因此EBV活化作为PTLD发生的独立危险因素，EBV-DNA载量可用于衡量机体免疫低下程度的指标之一，预测 PTLD发生的可能性，为PTLD抢先治疗提供依据。

由于allo-HSCT后PTLD发生与患者细胞免疫过度抑制导致EBV再活化相关，治疗的目的是恢复受者细胞免疫功能和去除EBV转化的B淋巴细胞，因此多采用在减少免疫抑制剂的基础上应用CD20单克隆抗体或联合T细胞输注（如DLI或EBV-CTL等）。本研究中29例PTLD患者接受了利妥昔单抗单药或联合静脉免疫球蛋白、DLI等方案治疗，治疗反应率达86.2％。EBV载量越高，治疗效果越差，可能与高病毒载量导致疾病进展迅速、利妥昔单抗治疗次数不足等有关。死亡患者大部分死于原发病复发、感染、重要脏器出血等，这部分患者EBV载量相对较低，因此存活与死亡患者间EBV载量无明显差异。由于利妥昔单抗的免疫抑制作用，长期严重的B细胞缺乏将导致细菌感染和死亡风险增加，联合静脉免疫球蛋白治疗可在一定程度上降低感染风险[24]。抢先治疗是减少PTLD发生的另一重要措施，利妥昔单抗（通常每周1～4剂）可产生88％的反应率，并明显降低PTLD的发生率[4]。由于并非所有EBV活化的患者都发生PTLD，如何选择合适的抢先治疗启动指证尚不明确。目前，不同研究单位间EBV检测方法、标本类型等不尽相同，因此各实验室结果无法比较。一些研究将血浆EBV-DNA 1×10^3^拷贝/ml[25]、全血4×10^4^拷贝/ml[26]、1×10^3^拷贝/10^6^外周血单个核细胞[27]作为利妥昔单抗抢先治疗的阈值。本研究结果表明全血EBV-DNA载量与PTLD发生高度相关，EBV-DNA载量处于10^4^～10^5^拷贝/ml时PTLD发生率与整体PTLD发生率大致相当，当大于此水平时PTLD发生率明显增高，EBV-DNA水平大于1×10^6^拷贝/ml时PTLD发生率高于30％，且EBV-DNA载量较低者利妥昔单抗治疗有效率较高，因此应用EBV-DNA载量作为预测PTLD发生和诊断PTLD的依据具有一定的意义。本研究结果提示可考虑将全血EBV-DNA 1×10^5^拷贝/ml作为抢先治疗预防PTLD的阈值。各移植中心采用统一的标准检测方法，制订统一的抢先治疗阈值对提高PTLD的治疗效果至关重要。

综上所述，PTLD是一组在免疫监视功能破坏及EBV感染的背景下产生的异质性肿瘤，进展快且预后较差。具有PTLD高危因素患者应密切监测EBV的再激活，根据EBV活化的程度预测和诊断PTLD的发生，并应用利妥昔单抗为主的方案进行抢先治疗，以降低PTLD发生风险和改善预后。
